# Multitask Learning Radiomics on Longitudinal Imaging to Predict Survival Outcomes following Risk-Adaptive Chemoradiation for Non-Small Cell Lung Cancer

**DOI:** 10.3390/cancers14051228

**Published:** 2022-02-26

**Authors:** Parisa Forouzannezhad, Dominic Maes, Daniel S. Hippe, Phawis Thammasorn, Reza Iranzad, Jie Han, Chunyan Duan, Xiao Liu, Shouyi Wang, W. Art Chaovalitwongse, Jing Zeng, Stephen R. Bowen

**Affiliations:** 1 Department of Radiation Oncology, School of Medicine, University of Washington, Seattle, WA 98195, USA; pforo003@uw.edu (P.F.); dmaes@uw.edu (D.M.); jzeng13@uw.edu (J.Z.); 2 Clinical Research Division, Fred Hutchinson Cancer Research Center, Seattle, WA 98109, USA; dhippe2@fredhutch.org; 3 Department of Industrial Engineering, University of Arkansas, Fayetteville, AR 72701, USA; pthammas@email.uark.edu (P.T.); riranzad@email.uark.edu (R.I.); xl027@uark.edu (X.L.); artchao@uark.edu (W.A.C.); 4 Department of Industrial, Manufacturing, and System Engineering, University of Texas, Arlington, TX 76019, USA; jie.han@mavs.uta.edu (J.H.); shouyiw@uta.edu (S.W.); 5 Department of Mechanical Engineering, Tongji University, Shanghai 200092, China; duanchunyan@tongji.edu.cn; 6 Department of Radiology, School of Medicine, University of Washington, Seattle, WA 98195, USA

**Keywords:** FDG-PET, CT, SPECT, multimodal imaging, lung cancer, radiomics, multitask regression, LASSO, gradient boosting, survival analysis

## Abstract

**Simple Summary:**

Personalized cancer treatment strategies, including risk-adaptive chemoradiation therapy based on medical imaging, seek to improve outcomes of patients with unresectable and locally advanced non-small cell lung cancer. Refining patient risk stratification relies on outcome prediction modeling based in part on information from different imaging modalities and imaging time points during and after treatment. Using prospectively collected longitudinal data from FDG-PET, CT, and perfusion SPECT images of patients enrolled on a clinical trial, our aim was to evaluate the utility of a multitask machine learning radiomics framework for survival outcome prediction. We found that multitask learning of FDG-PET radiomics on pretreatment and mid-treatment images achieved higher survival prediction concordance compared with single-task learning of other modalities and clinical benchmark models. Our multitask learning radiomics framework can be applied to other longitudinal imaging datasets, and, once validated, can strengthen clinical decision support for personalized and adaptive treatment courses.

**Abstract:**

Medical imaging provides quantitative and spatial information to evaluate treatment response in the management of patients with non-small cell lung cancer (NSCLC). High throughput extraction of radiomic features on these images can potentially phenotype tumors non-invasively and support risk stratification based on survival outcome prediction. The prognostic value of radiomics from different imaging modalities and time points prior to and during chemoradiation therapy of NSCLC, relative to conventional imaging biomarker or delta radiomics models, remains uncharacterized. We investigated the utility of multitask learning of multi-time point radiomic features, as opposed to single-task learning, for improving survival outcome prediction relative to conventional clinical imaging feature model benchmarks. Survival outcomes were prospectively collected for 45 patients with unresectable NSCLC enrolled on the FLARE-RT phase II trial of risk-adaptive chemoradiation and optional consolidation PD-L1 checkpoint blockade (NCT02773238). FDG-PET, CT, and perfusion SPECT imaging pretreatment and week 3 mid-treatment was performed and 110 IBSI-compliant pyradiomics shape-/intensity-/texture-based features from the metabolic tumor volume were extracted. Outcome modeling consisted of a fused Laplacian sparse group LASSO with component-wise gradient boosting survival regression in a multitask learning framework. Testing performance under stratified 10-fold cross-validation was evaluated for multitask learning radiomics of different imaging modalities and time points. Multitask learning models were benchmarked against conventional clinical imaging and delta radiomics models and evaluated with the concordance index (c-index) and index of prediction accuracy (IPA). FDG-PET radiomics had higher prognostic value for overall survival in test folds (c-index 0.71 [0.67, 0.75]) than CT radiomics (c-index 0.64 [0.60, 0.71]) or perfusion SPECT radiomics (c-index 0.60 [0.57, 0.63]). Multitask learning of pre-/mid-treatment FDG-PET radiomics (c-index 0.71 [0.67, 0.75]) outperformed benchmark clinical imaging (c-index 0.65 [0.59, 0.71]) and FDG-PET delta radiomics (c-index 0.52 [0.48, 0.58]) models. Similarly, the IPA for multitask learning FDG-PET radiomics (30%) was higher than clinical imaging (26%) and delta radiomics (15%) models. Radiomics models performed consistently under different voxel resampling conditions. Multitask learning radiomics for outcome modeling provides a clinical decision support platform that leverages longitudinal imaging information. This framework can reveal the relative importance of different imaging modalities and time points when designing risk-adaptive cancer treatment strategies.

## 1. Introduction

Cancer mortality and incidence remains significant with aging and population growth amid a multitude of risk factors, wherein lung cancer features high mortality and incidence rates [1]. Despite recent advances in treatment strategies, including combinations of surgery, chemotherapy, immunotherapy, and radiation therapy, median overall survival for patients with non-small cell lung cancer (NSCLC) remains poor and not all patients derive similar benefit. This highlights the importance of discovering and validating biomarkers that are both sensitive to treatment effects and predict outcomes following combination cancer therapies [2], enabling patient risk stratification for individualized treatment techniques. Risk stratification studies have focused on early detection and cancerous nodules classification [3,4,5], histologic subtype classification [6], prognosis after radiation therapy or surgery [7], prediction of lung toxicity after radiation therapy [8,9], and prediction of response to chemoradiation or immunotherapy [10,11,12], utilizing a variety of survival prediction modeling [10,11,12,13,14,15,16,17,18,19,20].

Biomarkers from quantitative medical imaging, such as positron emission tomography (PET), computed tomography (CT), and single-photon emission computerized tomography (SPECT), have been used to assess various components of cancer treatment response or risk of treatment-related side effects [21,22,23]. Among these imaging modalities, fluorodeoxyglucose (FDG)-PET/CT has been applied for quantitative assessment of early tumor response to lung cancer therapy and predicting survival outcomes [24]. High throughput radiomic extraction of advanced quantitative imaging features to define tumor characteristics related to intensity, shape, and texture of intratumor heterogeneity [25,26] has also shown promise in the prediction of treatment response and association to clinical outcomes [27,28,29,30]. Radiomics models have primarily utilized CT images, ranging from tumor/peritumoral lung parenchyma features with conventional Cox proportional hazard modeling of disease-free survival [31] and logistic regression modeling of chemotherapy response [10], to transfer learning of convolutional neural networks (CNN) to predict overall survival [12], and radiomic biomarkers of tumor mutational burden (TMB) for response prediction following immunotherapy [11]. However, the aforementioned studies were restricted to single imaging modalities—which may neglect information content from other modalities [10,14,15,32]—or required large data sets to train deep learning models, which may not be appropriate for smaller available data sets in early phase clinical trial settings [11,33].

Some studies have leveraged multimodal imaging or biomarker combinations from tissue and peripheral blood. Multimodal CT and PET imaging biomarker logistic regression modeling was developed to predict tumor response to radiotherapy [34], while combined CT imaging and genomics in a Cox model with elastic net regularization was used to predict post-surgical recurrence risk [35]. Clinical features were combined with MRI-based radiomics to predict histologic subtype from a nomogram, using logistic regression [36]. Neural layer fusion of features from clinical data, gene expression, and copy number alteration within multimodal graph neural networks (MGNN) was used for survival prediction [37]. However, these studies all relied on a single time point prior to treatment, which neglects patient-specific response information that can guide treatment adaptation strategies [16,17,18,19,20].

Multitask learning is a paradigm that leverages information and relationships from multiple related tasks for improved robustness of prediction performance [38,39], with broad applications in bioinformatics [40] using clinical data [41,42,43,44,45,46]. Multitask learning can be implemented as a feature learning approach, under which the prediction model can learn common features for all tasks [47,48], as a task clustering approach for model hyperparameter tuning [49], and simultaneous learning of pairwise task relations and model parameters [50]. Multiple tasks can be defined by predicting different outcomes for a single time point or predicting one outcome over time at multiple time points, which is especially pertinent in longitudinal studies. It remains unknown whether single- or multitask learning radiomics approaches can improve survival outcome prediction relative to published prognostic factors.

While most multitask learning considers multiple outcome variables for joint prediction, we propose to leverage multitask learning for novel multipoint survival probability prediction, which captures the nonlinear relationship of temporal information in longitudinal imaging data. In the context of a prospective phase II clinical trial of risk-adaptive chemoradiation therapy for unresectable NSCLC, this investigation sought to evaluate the utility of multitask learning for survival outcome modeling on longitudinal images of FDG-PET, CT, and perfusion SPECT. Our aim was to evaluate whether multitask learning of pretreatment and mid-treatment radiomic features can improve survival outcome prediction relative to benchmark models consisting of clinical imaging features or delta radiomics. We also investigated the effect of multimodality imaging combinations on survival outcome prediction. Our multitask learning approach seeks to:

- ingest feature spaces spanning multiple modalities;
- learn tasks jointly defined by the prediction of survival outcome continuously over time instead of prediction at a single time horizon;
- overcome missing and unbalanced data encountered in longitudinal datasets;
- incorporate a kernel-based model of nonlinear relationships between radiomics and survival outcomes beyond linear relationships;
- train efficiently on modest sample sizes from early phase clinical trial datasets that can robustly scale to larger datasets;
- guard against error propagation across feature space modalities.

## 2. Materials and Methods

### 2.1. Participants and Clinical Trial Protocol

Data in this study was prospectively collected on 45 patients with unresectable American Joint Committee on Cancer v7 stage IIB–IIIB non-small cell lung cancer and Eastern Cooperative Oncology Group performance status 0–1 enrolled on the phase II FLARE-RT clinical trial (NCT02773238). All patients were screened for trial eligibility based on strict inclusion and exclusion criteria, including adequate pulmonary function, renal function, and liver function. PET/CT and SPECT/CT imaging was performed 1–2 weeks prior to treatment start, and PET/CT imaging was repeated during week 3 to assess early treatment response. Patients received standardized 6 weeks of chemoradiation therapy that was risk-adapted based on FDG-PET response, with PET responders receiving 60 Gy in 30 fractions to planning target volumes and PET-non responders receiving 74 Gy in 30 fractions via concomitant dose boost over the final 15 fractions. Consolidation durvalumab anti-programmed death ligand 1 (PDL1) immune checkpoint inhibitor therapy was administered in patients who enrolled after this regimen became the standard of care following the PACIFIC trial [51,52]. Table 1 lists the demographic and clinical information of patients, which formed the basis for variables in the benchmark clinical imaging model.

### 2.2. Image Acquisition and Processing

**Figure 1:**
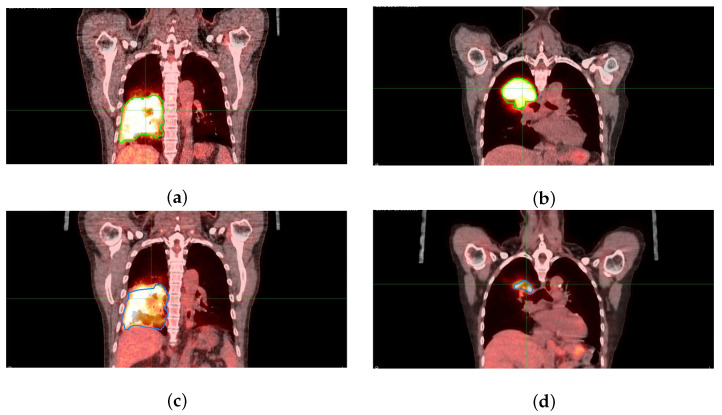
FDG-PET/CT images for an example PET non-responder patient (**a**,**c**) and PET responder patient (**b**,**d**), acquired pretreatment (**a**,**b**) and mid-treatment (**c**,**d**). Tumor volumes are displayed as blue/green contours.

Pretreatment and mid-treatment PET/CT imaging was performed in radiation treatment position with standardized imaging protocols on matching scanners and patient immobilization. Patients scanned on the GE Discovery STE (GE Healthcare, Waukesha, WI, USA) underwent acquisitions of 5 min per bed position while patients scanned on GE Discovery MI underwent acquisitions of 2.5 min per bed position due to differences in scanner sensitivity. PET ordered subset expectation maximization (OSEM) reconstruction parameters were harmonized between scanners to yield concordant quantitative images [53]. CT-based attenuation correction (CTAC) of the standardized uptake values (SUV) in quantitative PET was applied [54]. PET/CT images were rigidly aligned to the radiation therapy planning CT and corresponding dose distribution using mutual information in MIM 7.1 (MIM Software, Cleveland, OH, USA). Pretreatment [99 mTc] MAA perfusion SPECT/CT was performed on a Precedence (Philips Healthcare, Andover, MA) 16-slice scanner on all patients followed by correcting for attenuation, collimator–detector response, and scatter. SPECT image reconstruction was performed using ordered subset expectation maximization with spatial resolution recovery and a 10 mm cut-off filter. PET/CT and SPECT/CT images were co-registered to the reference planning CT via rigid alignment. Metabolic tumor volume (MTV) contours were prospectively delineated at each time point under the FLARE-RT trial protocol using a commercially validated semi-automatic gradient-based segmentation tool in MIM (PET Edge, MIM Software, Cleveland, OH, USA) with consensus from a multidisciplinary team, including a board-certified radiation oncologist. MTV contours defined in this way improved repeatability compared to manual contouring and reduced sensitivity to image reconstruction compared to fixed threshold contouring [53]. MTV contours were then propagated to all co-registered images. Figure 1 displays the pre-RT and mid-RT fused FDG-PET/CT images for an responder and non-responder to treatment from the FLARE-RT clinical trial.

### 2.3. Radiomic Feature Extraction

The MTV contour in DICOM RTstruct format along with the associated DICOM images at the native resolution (CT 0.8×0.8×2.5 mm^3^, FDG-PET 5.4×5.4×3.2 mm^3^, perfusion SPECT 4.6×4.6×4.6 mm^3^) were loaded separately for each modality using 3D slicer software into the imaging biomarker standardization initiative (IBSI) [55] compliant pyradiomics module [56]. No additional denoising of images was applied so as to preserve information content from clinical scanner protocols. All radiomic features were calculated over the 3D MTV mask volume and aggregated over voxels to report the average MTV feature values. Both native voxels and geometric mean resampled isotropic voxels (CT 1.2 mm, FDG-PET 4.5 mm, perfusion SPECT 4.6 mm) were utilized for texture feature calculation, in order to evaluate the reliability of our multitask learning radiomics framework [57,58,59,60,61,62]. Image intensities were discretized using standardized fixed bin width (FBW) of 25 HU, 25 CNTS, and 0.25 SUV for CT, SPECT, and PET, respectively, which promoted sufficient voxel sampling. Texture features were extracted without additional wavelet filtering to limit dimensionality. Of 110 total features, 75 texture features of gray-level run length (GLRLM), gray-level co-occurrence (GLCM), neighborhood gray tone difference (NGTDM), gray-level size zone (GLSZM), and gray-level dependence (GLDM) matrices, as well as 16 shape-based and 19 first-order intensity statistic-based features, were extracted.

### 2.4. Fused Laplacian Sparse Group LASSO (FLSGL)

In this longitudinal imaging biomarker study for cancer survival outcome modeling, the problem can be framed by multitask regression, either by predicting multiple outcomes or predicting an outcome at multiple time points. A multitask learning approach has been applied for the prediction of overall survival. Let us consider the input matrix of radiomic features for each modality as $X_{t}=\{x_{1},x_{2},\ldots ,x_{N}\}$ and target vector of overall survival as $y_{t}=\{y_{1},y_{2},\ldots ,y_{N}\}$; therefore, $X_{t}\in R^{N\times F}$ and $y_{t}\in R^{N}$, where *N* is the number of observations and *F* is the number of features at the time of t=1,2,…,T. It should be noted that all vectors are defined with lowercase letters, and matrices are defined with uppercase letters throughout this article. If the regression parameters across all tasks are considered as $\Phi \in R^{F\times T}$ matrix, then $\varphi \in R^{F}$ denotes the column of regression parameters of the task at the time, *t*. $W_{t}=\{w_{1},w_{2},\ldots ,w_{T}\}$ is the weight matrix at all time points. A local kernel-based smoothing approach [63] is used for local smoothing in order to minimize the regression error at each time point, and is associated with the task, *t*, and neighbor, $\varphi_{t}$. Thus, the approximation model can be determined as follows:

(1) $$\begin{array}{c} \hat{\varphi_{t}}=\sum_{\begin{array}{c} r=1\\ r\neq t \end{array}}^{T}w_{r,t}\varphi_{r},\;\;\;\;t=1,2,\ldots ,T \end{array}$$

where $w_{r,t}=\frac{K(\frac{r-t}{\sigma })}{\sum_{\begin{array}{c} r=1\\ r\neq t \end{array}}^{T}K(\frac{r-t}{\sigma })},\;\;\;\;r=1,2,\ldots ,T,r\neq t$.

Here σ is the bandwidth and *K* is the kernel matrix using the Gaussian kernel as

(2) $$\begin{array}{c} K=\frac{1}{\sigma \sqrt{2\pi }}exp\left(\frac{x^{2}}{2\sigma^{2}}\right) \end{array}$$

In Equation (1), the weights are defined by the Gaussian kernel where its bandwidth needs to be determined. A small value of σ leads to quick decay of the Gaussian curve, whereas a larger value promotes more gradual decay. We determined σ=1 as an appropriate default empirical value to be used in this study. On the other hand, the fused aspect of the model is obtained by adding sparsity on the matrix of residuals. The fused penalty or the transformation matrix as used in this study can be defined as $G\in R^{T\times T}$ in the term of P=ΦG as follows:

(3) $$\left[\begin{array}{cccc} \rho_{1} & \rho_{2} & \cdots & \rho_{T} \end{array}\right]={\left[\begin{array}{c} \varphi_{1}\\ \varphi_{2}\\ \vdots \\ \varphi_{T} \end{array}\right]}^{T}\left[\begin{array}{ccccc} I & -w_{\left|t-r\right|}I & -w_{\left|t-r\right|}I & \vdots & -w_{\left|t-r\right|}\\ -w_{\left|t-r\right|}I & I & \vdots & \vdots & -w_{\left|t-r\right|}I\\ \vdots & \vdots & \vdots & \vdots & \vdots \\ -w_{\left|t-r\right|}I & -w_{\left|t-r\right|}I & -w_{\left|t-r\right|}I & \cdots & I \end{array}\right]$$

The matrix of *G* includes the weights $w_{t,r}=w_{\left|t-r\right|}$, demonstrating the edges between the nodes *t* and *r*. Therefore, the solution for the multitask problem is to solve the following constrained optimization equation:

(4) $$\begin{array}{c} \begin{array}{c} \underset{\Phi ,P}{min}\sum_{t=1}^{T}\left|\right|y_{t}-X_{t}\varphi_{t}{\left|\right|}^{2}+H_{\beta 2}^{\beta 1}\left(\Phi \right)+{\beta 3\;||P||}_{1}\\ and\;\rho_{t}=\varphi_{t}-\sum_{\begin{array}{c} r=1\\ r\neq t \end{array}}^{T}w_{r,t}\varphi_{r} \end{array} \end{array}$$

where the columns of residuals, $\rho_{t}$, creates the matrix of residuals $P\in R^{F\times T}$, the β1, β2, and β3 are the regularization parameters, and $H_{\beta 2}^{\beta 1}\left(\Phi \right)={\beta 1\;||\left(\Phi \right)||}_{1}+{\beta 2\;||\left(\Phi \right)||}_{2,1}$ denotes the combination of penalties of the LASSO and the group LASSO. The group LASSO defined as ${\left||\left(\varphi \right)|\right|}_{2,1}=\sum_{i=1}^{F}\left|\right|\varphi_{i}\left|\right|$ considers the groups across all time points for each variable *i*, which allows sharing a common set of variables at each time point. In order to solve the optimization problem in Equation (4), which is ill-posed for direct optimization, an alternating direction method of multiplier (ADMM) is used. A detailed description of the multiblock ADMM steps for finding the matrix regression parameters, Φ can be found in [64]. Following this fused Laplacian sparse group LASSO (FLSGL) methodology, *M* modality-specific regression matrices are obtained to generate the *M* primary prediction of each target of $\hat{y}$ using $\hat{y}{{}_{M}}^{t}=X_{M}^{t}\times \Phi_{M}^{t}$ as well as to select the most important features. These outputs of selected features are then ensembled by the gradient boosting survival algorithm described in the next section.

### 2.5. Component-Wise Gradient Boosting Survival Analysis (CWGBS)

To account for a time-dependent outcome target variable with right censoring, a component-wise gradient boosting survival (CWGBS) approach was employed. Survival analysis is a series of statistical procedures which considers the time until an event occurs [65]. Right-censoring in survival analysis occurs when there is finite survival time information about each individual, who will each have variable duration of follow-up intervals over which an event may or may not occur. One can leverage a gradient boosting survival model that combines the prediction of multiple weak learners in an additive manners to achieve a powerful model. The overall model of the boosting algorithm can be defined as Equation (5).

(5) $$\begin{array}{c} {\hat{u}}_{V,X}=\underset{u}{argmin}\sum_{i=1}^{n}{\left(V_{i}-g\left(X_{i}|u\right)\right)}^{2} \end{array}$$

where N > 0 is the number of base or weak learners, *V* defines a pseudo-response variable, $u_{V,X}$ is a parameters vector, and the base learner regressing *v* on the covariates *X* is indicated by $g(.|u_{V,X})$. Since the provided least-squares problem cannot be solved for fitting the base learner due to dependency of $V_{i}$ to the censored $Y_{i}$, the weighted least squares of ${\tilde{u}}_{\tilde{V},X}$ is computed using the pseudo-responses (Equation (6)) follow by fitting the base learner $g(.|u_{V,X})$ to the new $v_{i}$.

(6) $$\begin{array}{c} {\tilde{V}}_{i}=-\frac{\partial L({\tilde{Y}}_{i},\varphi )}{\partial \varphi }|\varphi ={\hat{f}}_{m}\left(X_{i}\right) \end{array}$$

where $\hat{f}$ is the estimation of regression function, *L* is the loss function, and φ is the candidate estimators for the regression function *f*. Details of the mathematical formalism are described in [66]. Here, the CWGBS algorithm uses the partial likelihood loss of Cox’s proportional hazards model (coxph) as the loss function and component-wise least squares as the base learner that fits a regression tree of selected features of different modalities in the last step at each stage on the negative gradient of the loss function. The CWGBS output generates the predicted overall survival time as well as the probability of survival for each patient.

**Figure 2:**
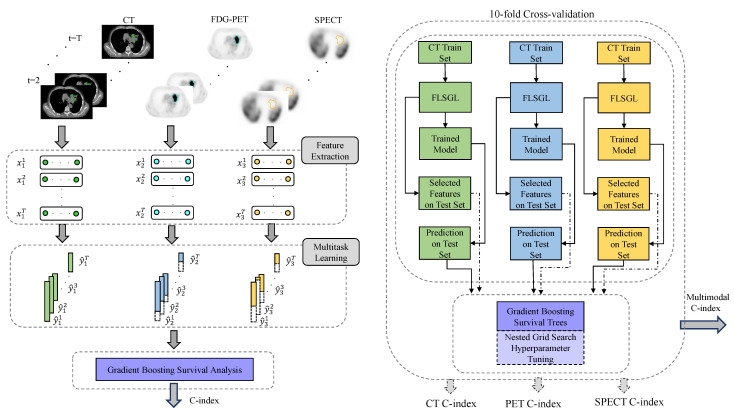
Overall schematic of survival outcome prediction pipeline using multitask feature selection across time points from single/multimodality radiomics (**left**) and steps inside the stratified cross-validation folds for multitask and gradient boosting survival (**right**). Note that feature selection and nested grid search for hyperparameter tuning were constrained to training folds and blinded to test folds, in order to prevent data leakage for unbiased performance evaluation.

The overall schematic of the multitask single/multimodality pipeline for prediction of survival outcomes is depicted in Figure 2. The multitask learning pipeline consisted of stratified 10-fold cross-validation repeated iteratively 15 times for different random seeds to ensure that the prevalence of overall survival events was similar in training and testing subsets, as well as to remove any bias in selecting training and testing subsets. The hyperparameters of CWGBS, including the number of estimators and the loss function type, as well as hyperparameters of the FLSGL feature selection algorithm, were optimized by nested grid search in python using the Scikit-learn library within the training sets and blinded to testing sets in order to prevent data leakage. Tuned hyperparameters for CWGBS were constrained to no more than 15 base weak learners (estimators) for the Cox proportional hazard (Coxph) loss function with a learning rate of 1. Tuned FLSGL hyperparameters included σ=1, δ=10, $\beta_{1}=4.8$, $\beta_{2}=2.2$, and $\beta_{3}=4.2$ using the nested grid search in the range of [1, 5].

## 3. Results

**Figure 3:**
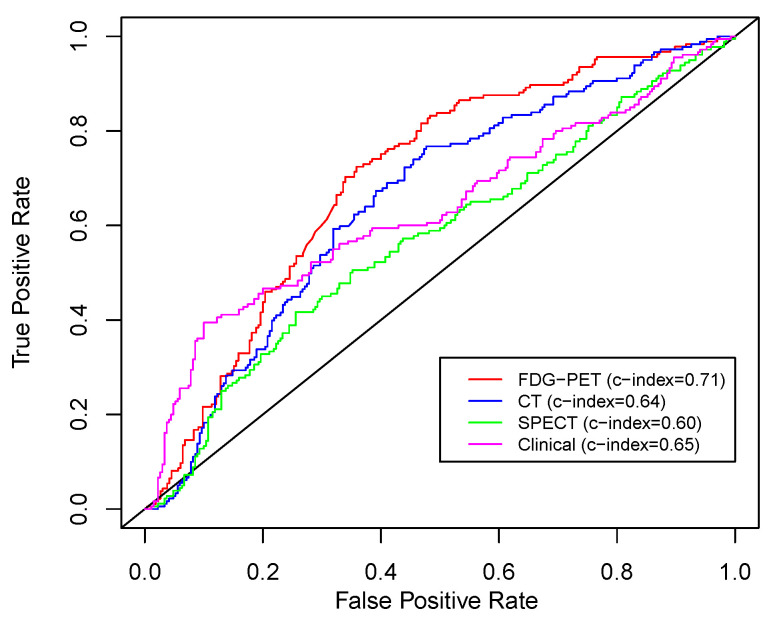
Receiver operating characteristic (ROC) curves and c-index values for different modalities.

Overall survival prediction performance of the models in test folds, based on concordance index (c-index) along with 95% confidence intervals, is reported in Table 2 for single task learning of different modalities compared to multitask learning of pre-/mid-treatment time points. Table 2 reports non-parametric Friedman ANOVA statistical testing of multitask versus single task learning models, as well as Wilcoxon signed rank statistical testing of radiomics model performance in comparison to the benchmark clinical imaging model. From the patient characteristics listed in Table 1, the benchmark clinical imaging model included LASSO-selected CT planning target volume, FDG-PET SUVmax, FDG-PET metabolic tumor volume, and FDG-PET total lesion glycolysis. As seen in Table 2, the c-index of the multitask pre-/mid-RT FDG-PET radiomics model is significantly higher than the c-index of the benchmark clinical imaging model. In addition to c-index, the index of prediction accuracy (IPA) was computed for the multitask learning models. IPA combines calibration and discrimination in one performance evaluation metric by rescaling the Brier score, which enhances interpretability by adjusting for a reference model [67]. A 2-year time horizon and reference (null) model of Kaplan–Meier were considered for calculating IPA. We obtained a higher IPA of 30% for FDG-PET multitask learning, compared to 26% for the clinical imaging benchmark and 15% for the delta radiomics benchmark, which was consistent with the c-index results. By contrast, CT and SPECT radiomics achieved lower performance relative to FDG-PET radiomics. Figure 3 visualizes the receiver operating characteristic (ROC) curves along with the c-index for different modalities.

Table 3 summarizes overall survival prediction performance for the proposed model of a single time point or two time points for different combinations of modalities. Here, in the first step, the FLSGL model was applied on each modality at different time points (either single or multitask at each column). Then the result of each modality obtained was ensembled using the CWGBS model for different multimodality combinations at each row. Table 3 demonstrates that combining other modalities with FDG-PET does not improve the prediction results either on the pre-RT or mid-RT time point. However, Friedman ANOVA testing reveals that multitask learning of pre-RT/mid-RT achieves higher model concordance for each multimodality combination relative to single task learning. This mirrors the improved prediction performance of multitask learning of individual modalities in Table 2.

**Figure 4:**
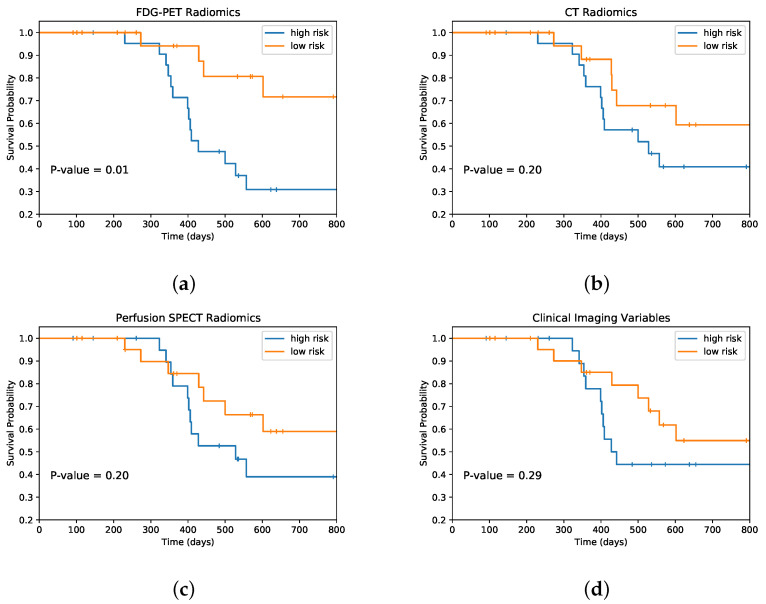
Kaplan–Meier curves of overall survival in test folds stratified by high-risk (>median prediction) versus low-risk (<median prediction) groups with models using the (**a**) FDG-PET, (**b**) CT, (**c**) perfusion SPECT radiomic features, and (**d**) clinical imaging variables.

**Figure 5:**
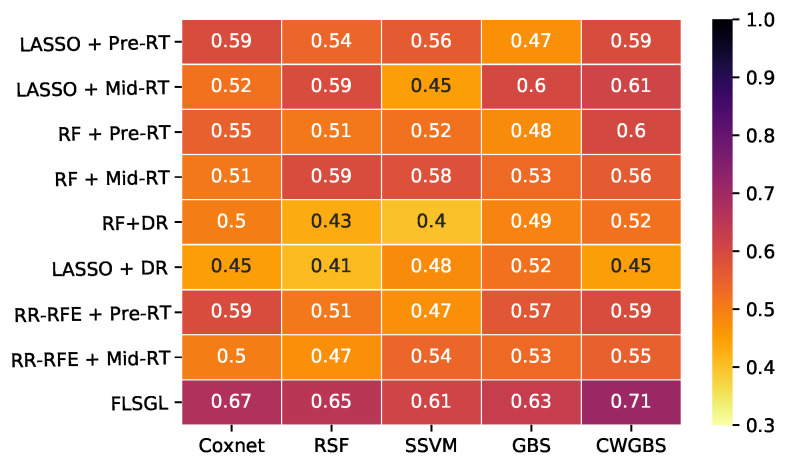
Heatmap of c-index values of overall survival prediction for different feature selection and survival analysis algorithms using FDG-PET radiomics (DR—delta radiomics; Coxnet—Cox net survival model; RR-RFE—ridge regression recursive feature elimination; RF—random forest; RSF—random survival forest; GBS—gradient boosting survival; SSVM—survival support vector machine; GBS—gradient boosting survival).

**Figure 6:**
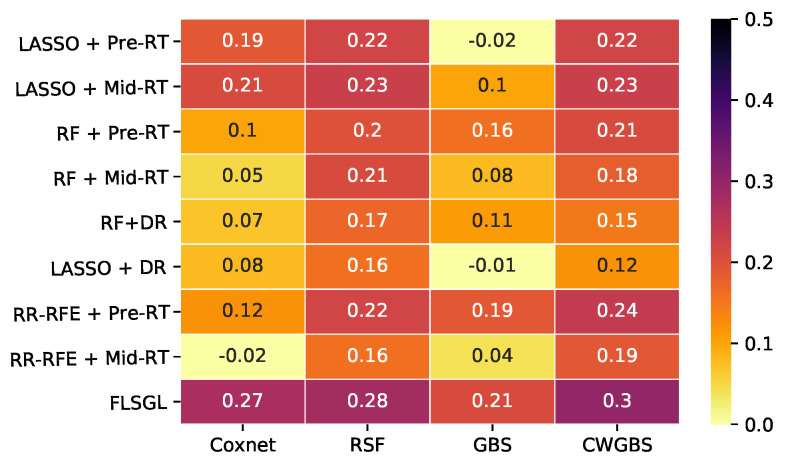
Heatmap of IPA values of overall survival prediction for different feature selection and survival analysis algorithms using FDG-PET radiomics (DR—delta radiomics; Coxnet—Cox net survival model; RR-RFE—ridge regression recursive feature elimination; RF—random forest; RSF—random survival forest; GBS—gradient boosting survival; SSVM—survival support vector machine; GBS—gradient boosting survival).

Figure 4 displays the Kaplan–Meier curves for the test folds stratified into 2 groups of high risk (above median prediction) and low risk (below median prediction) for each modality of FDG-PET, CT, and perfusion SPECT radiomic features, along with benchmark clinical imaging variables. Statistically significant stratification of low-risk versus high-risk groups was achieved using FDG-PET radiomics (log rank *p* = 0.01). Results in Figure 3 and Figure 4 are generated based on aggregating test samples across the stratified 10-fold cross-validation and 15 iterative resamplings. Table 4 and Figure 5 and Figure 6 summarize the performance of different learning combinations, including our multitask learning framework compared to other machine learning approaches in terms of c-index and IPA. Of note, delta radiomics models [68,69], in which feature differences between time points are used as predictors, showed lower performance as compared to our proposed multitask learning approach, which combines fused Laplacian sparse group LASSO feature selection with component-wise gradient boosting survival trees.

Supplemental results based on texture features extracted from geometric mean resampled isotropic voxels are reported in Supplementary Tables S1–S3 and Figures S1–S4. The isotropic radiomic model performance trends were consistent with those of models based on texture features from native voxels following standardized clinical trial imaging protocols.

## 4. Discussion

We developed and implemented a novel multitask learning radiomics framework over multiple imaging time points using the fused Laplacian sparse group LASSO (FLSGL) kernel-based algorithm that can capture non-linear associations to clinical outcomes. The framework was applied to survival outcome modeling in a cohort of patients with unresectable non-small cell lung cancer enrolled on the FLARE-RT phase II clinical trial, from which CT, FDG-PET, and perfusion SPECT radiomic features at pre- and mid-treatment time points were extracted. Stratified 10-fold cross-validation with 15 iterative resamplings was utilized to ensure consistent survival event proportionality across training/testing folds and guard against overfitting when reporting test set performance. In addition, an ensemble approach using component-wise gradient boosting survival (CWGBS) was applied to the primary predictions of separate-modality regressions to improve the overall estimation of prediction. Higher concordance was achieved for prediction of overall survival using PET radiomic features relative to a benchmark model using clinical imaging factors. Moreover, multitask learning of pretreatment and mid-treatment time points jointly resulted in improved survival prediction performance compared with delta radiomics modeling between time points, which highlights the power and flexibility of multitask learning. Radiomics model performance trends between multitask learning and single-task learning were consistent under different voxel resampling conditions. We observed differences in the importance of radiomic features across modalities. Shape and intensity features, such as volume and uni-dimensional length measures, were most frequently selected as a percentage of all radiomic features from FDG-PET (89%) and CT (64%). These pyradiomics features included voxel volume, major axis length, and total energy. By contrast, 86% of the most frequently selected SPECT radiomic features were from texture feature families.

Limited studies have investigated multitask learning in cancer survival outcome modeling and associations of medical imaging features to clinical factors [32,43,44,45,70,71]. Fan et al. mapped the radiomic features of MRI to tumor proliferation Ki-67 and tumor grade using a multitask learning to enhance prediction performance of breast cancer with the assumption of sharing common patterns of different source of features [70]. The same approach of joint prediction using a multiobjective Bayesian network for radiation pneumonitis and tumor local control was utilized in NSCLC radiotherapy [71]. In addition, Zhang et al. proposed a 2-layer pyramid network to first extract 737 radiomic features from CT images along with a LASSO feature selection and a joint multitask to learn simultaneously from correlated tasks of survival and prognosis prediction of gastric cancer [43]. Another investigation developed a combination of deep learning and radiomics to jointly classify atypical adenomatous hyperplasia/adenocarcinoma and minimally invasive adenocarcinoma, non-invasive adenocarcinomas, and invasive adenocarcinomas [32]. However, these studies considered tasks at a single time point and obtained shared information of correlated tasks to improve the prediction performance. They did not incorporate patient-specific response for treatment adaption during the course of therapy at multiple time points, which limits their application in the context of longitudinal studies.

Chi et al. proposed semi-supervised multitask learning for survival analysis on four different cancer data sets [44]. They applied model prediction error by randomly adding noise to each feature in order to obtain the feature importance and a deep learning-based model to transform the time-dependent analysis into multitask learning, which includes survival probability prediction at multiple time points. In this designed structure, a semi-supervised loss is used to deal with censored or unlabeled data, logarithmic loss for the binary classification of labeled data, and a ranking loss to deal with the prior knowledge of the non-increasing survival probability trend. They considered the survival time as multiple discrete time points rather than a continuous variable and applied the multitask learning on survival time points, with confined learning of a single imaging modality. In addition, the aforementioned studies performed training on large datasets, which may not readily translate to the setting of smaller sample sizes prevalent in early phase clinical trials. Therefore, an efficient learning method with unbiased performance evaluation is crucial to secondary analyses of early phase prospective clinical trials with limited patient sample sizes.

Our novel multimodal multitask learning framework can be applied on different dataset sizes with different therapy duration at multiple time points. To the best of our knowledge, this is the first multitask learning study to predict the survival outcomes for patients with unresectable NSCLC in the context of an early phase clinical trial. Multitask learning radiomics extracts greater information content across longitudinal imaging than delta radiomics, including optimizing combinations of imaging modalities and time points. Delta radiomics approaches for predictive modeling have been proposed by several studies [69,72,73,74]. Fave et al. used delta radiomics to investigate the prognostic improvement for NSCLC of 107 patients [69]. Applying multivariate models, delta radiomics improved the c-index of overall survival prediction and changed significantly during the treatment. Another study found that the combination of conventional radiomic features with delta radiomics in lung cancer screening can improve pulmonary nodule malignancy prediction [74]. We compared the PET delta radiomics models for overall survival prediction against multitask learning models of pre-/mid-treatment PET radiomics and observed significant improvement in performance with multitask learning. This highlights the effectiveness of capturing the nonlinear relationship of radiomic features and survival outcome jointly across time points.

Outcome modeling can enable identification of prognostic signatures and a means of risk stratifying patients for increasingly precise combinations of cancer therapies. Prognostic decision making usually depends on multifactorial aggregation of information, including but not limited to histologic, genetic, and molecular testing along with clinician heuristic experience [75]. Prediction of survival using non-invasive imaging features can facilitate risk stratification. Identifying patients at high risk for disease progression and poor survival prognosis early during the course of treatment can empower treatment intensification strategies, including radiation dose escalation, radiosensitizing chemotherapies, and immune-modulating therapies. In this study, survival outcome modeling with multitask learning radiomics can stratify patients to support decisions related to personalized adaptive cancer therapies. The proposed model is able to stratify high-risk and low-risk groups (as shown in Figure 4) based on FDG-PET radiomic features. Moreover, multitask learning radiomics can be applied to other clinical settings with longitudinal imaging, including CT, PET, or MRI for response assessment, daily cone beam CT or MRI for adaptive radiation therapy, and theranostic strategies utilizing both serial PET and SPECT imaging. The multitask learning framework can reveal the relative importance of different imaging modalities and different imagine time points, which may be leveraged when designing imaging components of future clinical trials. This may include longitudinal imaging to assess novel strategies combining anti-angiogenic therapy and immunotherapy that seek to alter the tumor microenvironment towards favorable response patterns [76]. Further investigation with multitask learning to elucidate the biological meaning of radiomics in response to therapy is warranted [77].

Despite the potential of multitask learning to improve survival outcome modeling relative to single task learning and clinical benchmarks, this study has several limitations. One of the major limitations is a small number of participants due to constraints from an early phase single-institution clinical trial. Modest sample sizes in early phase trials present challenges to the model generalizability, requiring internal validation techniques (cross-validation, bootstrapping, optimism adjustment) rather than testing on external validation datasets. From this early phase investigation, validation of the proposed multitask learning radiomics framework on a larger independent cohort of patients is a focus of future work. We observed that adding information from CT or SPECT modalities to FDG-PET did not improve outcome models. Although the multimodality imaging models did not enhance the overall survival prediction performance, multitask learning did improve survival outcome prediction for multimodality combinations relative to single task learning. Other imaging modalities in our cohort may not have contributed relevant prognostic information beyond PET due to the lack of contrast on CT images acquired in the PET/CT exams, which differ from contrast enhanced diagnostic CT exams. Likewise, low spatial resolution of perfusion SPECT imaging may have limited the extraction of relevant features from tumor regions. Furthermore, while the current study demonstrates improvement of multitask learning in overall survival prediction, each of the survival outcomes are considered separately across multiple time points. Model prediction performance may be improved by considering multiple survival outcomes, such as overall survival and disease-free survival as multitask learning targets over time continuously. Our prediction results rely on only two imaging time points separated by three weeks of time for multitask learning. Adding imaging time points after treatment with larger time intervals would enhance survival outcome model prediction performance, though time points during treatment promote earlier intervention and treatment adaptation. Finally, other longitudinal biomarkers beyond imaging derived from tissue and blood assays can be integrated into the multitask learning framework in the future.

## 5. Conclusions

In this investigation, we evaluated the radiomic features of FDG-PET, CT, and SPECT imaging for prediction of overall survival in patients with non-small cell lung cancer. A multitask learning approach considering pre-/mid treatment time points combined with a gradient boosting survival network has been applied to different imaging modalities. FDG-PET radiomics carried greater prognostic value than CT or perfusion SPECT radiomics in our clinical trial cohort. Multitask learning models of longitudinal FDG-PET outperformed benchmark clinical imaging and delta radiomics models. Multitask learning of multimodality radiomics should be further investigated and validated for outcome modeling, with the potential to provide clinical decision support during risk-adaptive cancer therapy.

## Figures and Tables

**Table 1:** Demographic and clinical information of the participants in the FLARE-RT clinical trial.

Characteristics		Value *
Age		63 (34–78)
Gender		
	Female	25 (56%)
	Male	20 (44%)
Clinical Stage (AJCCv7)		
	IIB	2 (4%)
	IIIA	23 (51%)
	IIIB	15 (33%)
	N2 Recurrence	5 (11%)
Histology		
	Squamous cell carcinoma	14 (31%)
	Adenocarcinoma	29 (64%)
	other	2 (4%)
Radiation therapy		
	Proton scanning beam therapy	23 (51%)
	X-ray radiotherapy (IMRT/VMAT)	22 (49%)
Chemotherapy		
	Carboplatin + paclitaxel	25 (56%)
	Cisplatin + etoposide	11 (24%)
	Other platinum doublet	9 (20%)
PD-L1 tumor proportion score		
	>50%	6 (13%)
	1–49%	7 (16%)
	<1%	7 (16%)
	Unknown	25 (56%)
Mid-PET Response		
	Responder	29 (64%)
	Non-responder	16 (36%)
Mid-PET PERCIST 1.0		
	Partial metabolic responder	27 (60%)
	Stable metabolic disease	17 (38%)
	Progressive metabolic disease	1 (2%)

* Values represent the number of patients (%) or median (range) for all attributes.

**Table 2:** Prediction performance of overall survival for the proposed model of FLSGL combined with CWGBS using a single time point or multiple time points for each modality. Values represent c-index (95% confidence interval) with *p*-values of the Friedman ANOVA test for multitask versus single task learning, as well as *p*-values of the Wilcoxon signed rank test for multitask learning of each modality relative to the benchmark model using clinical variables.

Modality	Single Task (Pre-RT)	Single Task (Mid-RT)	Multitask (Pre-RT/Mid-RT)	Friedman *p*-Value	Wilcoxon Signed Rank *p*
FDG-PET	0.66 (0.61–0.70)	0.63 (0.56–0.67)	0.71 (0.67–0.75)	<0.01	0.02
CT	0.56 (0.52–0.61)	0.64 (0.60–0.71)	0.64 (0.59–0.72)	0.01	0.23
SPECT *	0.60 (0.57–0.63)	-	-	-	0.20
Clinical Variables	0.63 (0.58–0.70)	0.62 (0.56–0.67)	0.65 (0.61–0.71)	0.06	reference

* No perfusion SPECT images acquired mid-RT.

**Table 3:** Prediction performance of overall survival in terms of c-index for the proposed model of a single time point or multiple time points for the combination of modalities. Here, FLSGL was applied on each modality at single-/multi-time points separately and results (each row) were ensembled using CWGBS at single-/multi-time points for different multimodality combinations. *p*-values of the Friedman ANOVA test are reported for each modality combination between multitask and single-task learning time points.

Modalities	Single Task (Pre-RT)	Single Task (Mid-RT)	Multitask (Pre-RT/Mid-RT)	Friedman *p*-Value
FDG-PET + CT	0.62 (0.58–0.66)	0.63 (0.59–0.68)	0.66 (0.63–0.70)	0.03
FDG-PET + SPECT	0.59 (0.56–0.63)	0.63 (0.56–0.67)	0.65 (0.61–0.69)	0.01
FDG-PET + Clinical Variables	0.63 (0.58–0.68)	0.60 (0.55–0.66)	0.67 (0.64–0.72)	<0.01
FDG-PET + CT + SPECT	0.57 (0.54–0.61)	0.60 (0.56–0.65)	0.63 (0.59–0.67)	<0.01
FDG-PET + CT + SPECT + Clinical Variables	0.57 (0.53–0.61)	0.61 (0.55–0.66)	0.62 (0.57–0.67)	0.01

**Table 4:** Comparison of FDG PET radiomics overall survival prediction models between the proposed FLSGL and CWGBS with different feature selection and survival regression models (DR—delta radiomics; Coxnet—Cox net survival model; RR-RFE—ridge regression recursive feature elimination; RF—random forest; FLSGL—fused Laplacian sparse group LASSO; RSF—random survival forest; GBS—gradient boosting survival; SSVM—survival support vector machine; CWGBS—component-wise gradient boosting survival). Bolded values denote highest level of performance.

Feature Selection	Survival Analysis	Time Points	No. of Features	C-Index (95% Confidence Interval)	IPA (%)
LASSO	CWGBS	Pre-RT	3–7	0.59 (0.55–0.66)	22
LASSO+DR	CWGBS	pre-/mid-RT	1–6	0.45 (0.40–0.51)	12
RF+DR	CWGBS	pre-/mid-RT	2–10	0.52 (0.48–0.58)	15
RR-RFE	CWGBS	Pre-RT	2–7	0.54 (0.51–0.60)	24
RF	CWGBS	Pre-RT	3–12	0.61 (0.55–0.66)	21
FLSGL	RSF	pre-/mid-RT	1–5	0.65 (0.60–0.70)	28
FLSGL	Coxnet	pre-/mid-RT	1–5	0.67 (0.63–0.72)	27
FLSGL	SSVM	pre-/mid-RT	1–5	0.62 (0.59–0.69)	- *
FLSGL	GBS	pre-/mid-RT	1–5	0.63 (0.58–0.68)	21
**FLSGL**	**CWGBS**	**pre-/mid-RT**	1–5	**0.71 (0.67–0.75)**	**30**

* Brier score-derived IPA is not calculated as SSVM does not generate predicted probability.

## Data Availability

The data from the FLARE-RT clinical trial (NCT02773238) will be made available on The Cancer Imaging Archive (TCIA) upon trial completion and reporting of mature results.

## Supplementary Materials

The following supporting information can be downloaded at: https://www.mdpi.com/article/10.3390/cancers14051228/s1, Table S1: Prediction performance of overall survival for the proposed model of FLSGL combined with CWGBS using a single time point or multiple time points for each modality. Values represent c-index (95% Confidence interval) with *p*-values of the Friedman ANOVA test for multitask versus single task learning time points, as well as *p*-values of the Wilcoxon signed rank test for multitask learning of each modality relative to the benchmark model using clinical variables; Table S2: Prediction performance of overall survival in terms of c-index for the proposed model of a single time point or multiple time points for the combination of modalities. Here, FLSGL was applied on each modality at single/multi-timepoints separately and results (each row) were ensembled using CWGBS at single/multi timepoints for different multimodality combinations. *p*-values of the Friedman ANOVA test are reported for each modality combination between multitask and single task learning time points; Table S3: Comparison of FDG PET radiomics overall survival prediction models between the proposed FLSGL and CWGBS with different feature selection and survival regression models (DR: Delta Radiomics, Coxnet: Cox Net Survival Model, RR-RFE: Ridge Regression Recursive Feature Elimination, RF: Random Forest, FLSGL: Fused Laplacian Sparse Group LASSO, RSF: Random Survival Forest, GBS: Gradient Boosting Survival, SSVM: Survival Support Vector Machine, and CWGBS: Component-Wise Gradient Boosting Survival); Figure S1: Receiver-operating Characteristic (ROC) curves and c-index values for different modalities; Figure S2: Kaplan-Meier curves of overall survival in test folds stratified by high risk (>median prediction) versus low risk (<median prediction) groups with models using the (a) FDG-PET, (b) CT, (c) SPECT radiomic features, and (d) clinical-imaging variables; Figure S3: Heatmap of c-index values of overall survival prediction for different feature selection and survival analysis algorithms using FDG-PET radiomics (DR: Delta Radiomics, Cox: Cox Net Survival Model, RR-RFE: Ridge Regression Recursive Feature Elimination, RF: Random Forest, FLSGL: Fused Laplacian Sparse Group LASSO, RSF: Random Survival Forest, GBS: Gradient Boosting Survival, SSVM: Survival Support Vector Machine, and CWGBS: Component-Wise Gradient Boosting Survival); Figure S4: Heatmap of IPA values of overall survival prediction for different feature selection and survival analysis algorithms using FDG-PET radiomics (DR: Delta Radiomics, Cox: Cox Net Survival Model, RR-RFE: Ridge Regression Recursive Feature Elimination, RF: Random Forest, FLSGL: Fused Laplacian Sparse Group LASSO, RSF: Random Survival Forest, GBS: Gradient Boosting Survival, and CWGBS: Component-Wise Gradient Boosting Survival).
