# Supplementary material for: Multitask Learning Radiomics on Longitudinal Imaging to Predict Survival Outcomes following Risk-Adaptive Chemoradiation for Non-Small Cell Lung Cancer
Source: Cancers (Basel). 2022 Feb 26;14(5):1228. doi: 10.3390/cancers14051228 (PMC8909466; doi:10.3390/cancers14051228)
Supplement: Supplementary file 1 [file cancers-14-01228-s001.zip › cancers-1527098-supplementary.pdf]

Supplemental Materials: Isotropic voxel-based radiomic models

Table S1: Prediction performance of overall survival for the proposed model of FLSGL combined with CWGBS using a single time point or multiple time points for each modality. Values represent c-index (95% Confidence interval) with p-values of the Friedman ANOVA test for multitask versus single task learning time points, as well as p-values of the Wilcoxon signed rank test for multitask learning of each modality relative to the benchmark model using clinical variables.

Modality	Single task (Pre-RT)	Single task (Mid-RT)	Multitask (Pre-RT/Mid-RT)	Friedman p-value	Wilcoxon signed rank p
FDG-PET	0.64 (0.59-0.69)	0.59 (0.54-0.64)	0.69 (0.65-0.73)	<0.01	0.05
CT	0.56 (0.52-0.59)	0.66 (0.61-0.72)	0.66 (0.62-0.70)	0.01	0.77
SPECT*	0.60 (0.57 - 0.63)	-	-	-	0.20
Clinical Variables	0.63 (0.58 - 0.70)	0.62 (0.56 - 0.67)	0.65 (0.61 - 0.71)	0.06	reference

*No perfusion SPECT images acquired mid-RT

Table S2: Prediction performance of overall survival in terms of c-index for the proposed model of a single time point or multiple time points for the combination of modalities. Here, FLSGL was applied on each modality at single/multi-timepoints separately and results (each row) were ensembled using CWGBS at single/multi timepoints for different multimodality combinations. P-values of the Friedman ANOVA test are reported for each modality combination between multitask and single task learning time points.

Modalities	Single task (Pre-RT)	Single task (Mid-RT)	Multitask (Pre-RT/Mid-RT)	Friedman p-value
FDG-PET + CT	0.61 (0.58-0.64)	0.64 (0.60-0.69)	0.66 (0.63-0.70)	0.01
FDG-PET + SPECT	0.60 (0.56-0.64)	0.59 (0.54-0.64)	0.64 (0.60-0.68)	0.01
FDG-PET + Clinical Variables	0.63 (0.58-0.68)	0.59 (0.54-0.64)	0.66 (0.62–0.70)	0.03
FDG-PET + CT + SPECT	0.57 (0.54-0.60)	0.64 (0.60-0.69)	0.64 (0.59-0.68)	<0.01
FDG-PET + CT + SPECT + Clinical Variables	0.57 (0.53-0.62)	0.57 (0.52-0.63)	0.62 (0.57-0.67)	0.01

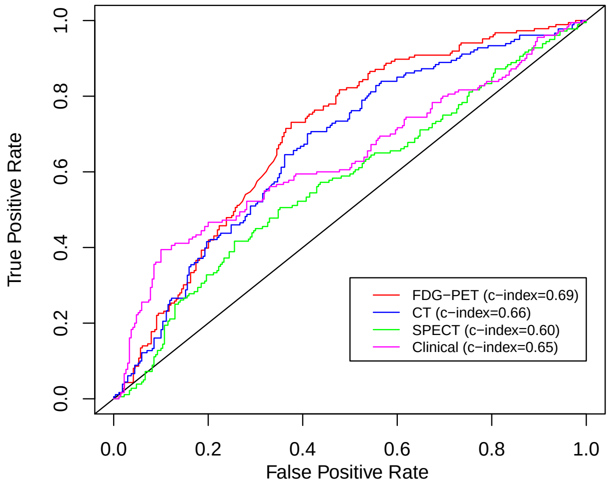

Figure S1. Receiver-operating Characteristic (ROC) curves and c-index values for different modalities.

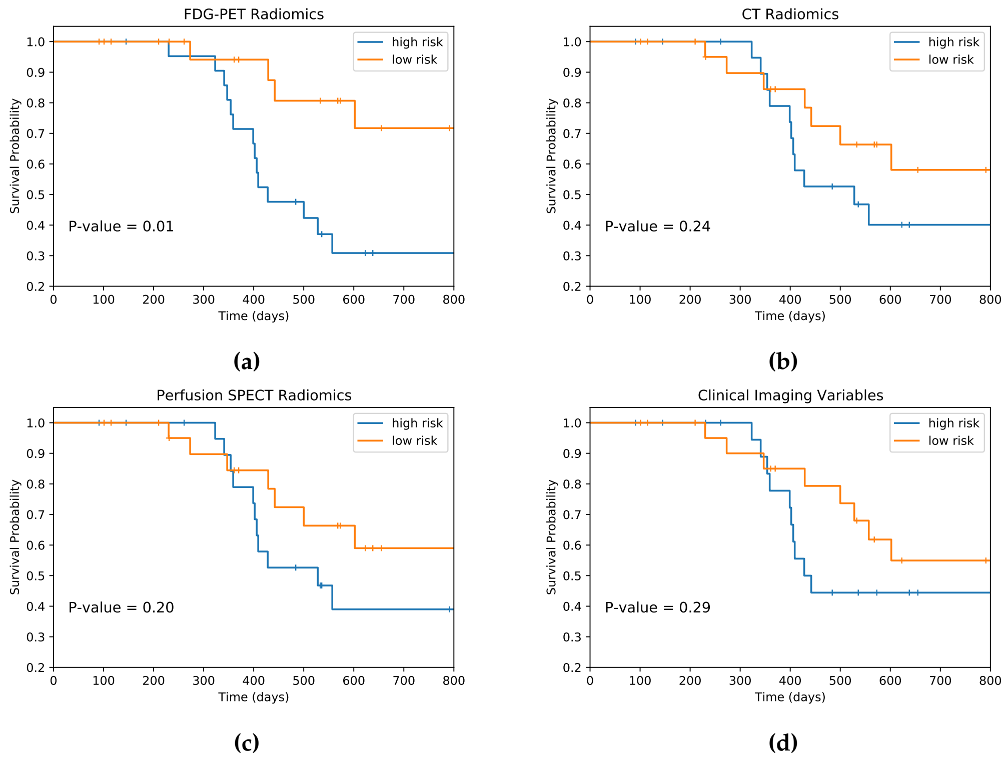

Figure S2. Kaplan-Meier curves of overall survival in test folds stratified by high risk (> median prediction) versus low risk (< median prediction) groups with models using the a) FDG-PET b) CT, c) SPECT radiomic features, and d) clinical-imaging variables.

Table S3: Comparison of FDG PET radiomics overall survival prediction models between the proposed FLSGL and CWGBS with different feature selection and survival regression models. (DR: Delta Radiomics, Coxnet: Cox Net Survival Model, RR-RFE: Ridge Regression Recursive Feature Elimination, RF: Random Forest, FLSGL: Fused Laplacian Sparse Group Lasso, RSF: Random Survival Forest, GBS: Gradient Boosting Survival, SSVM: Survival Support Vector Machine, and CWGBS: Component-Wise Gradient Boosting Survival)

Feature Selection	Survival Analysis	Time Points	No. of Features	C-index (95% confidence interval)	IPA (%)
LASSO	CWGBS	Pre-RT	2-7	0.55 (0.51 - 0.61)	19
LASSO+DR	CWGBS	Pre/Mid-RT	1-6	0.45 (0.41 - 0.58)	-12
RF+DR	CWGBS	Pre/Mid-RT	4-12	0.54 (0.50 - 0.59)	13
RR-RFE	CWGBS	Pre-RT	10-15	0.61 (0.56 - 0.65)	23
RF	CWGBS	Pre-RT	4-14	0.56 (0.51 - 0.60)	19
FLSGL	RSF	Pre/Mid-RT	1-6	0.66 (0.60 - 0.71)	29
FLSGL	Coxnet	Pre/Mid-RT	1-5	0.67 (0.63 - 0.72)	29
FLSGL	SSVM	Pre/Mid-RT	1-5	0.62 (0.59 - 0.69)	-*
FLSGL	GBS	Pre/Mid-RT	1-5	0.59 (0.58 - 0.64)	13
**FLSGL**	**CWGBS**	**Pre/Mid-RT**	**1-5**	**0.69 (0.65 – 0.73)**	**30**

*Brier score-derived IPA is not calculated as SSVM does not generate predicted probability

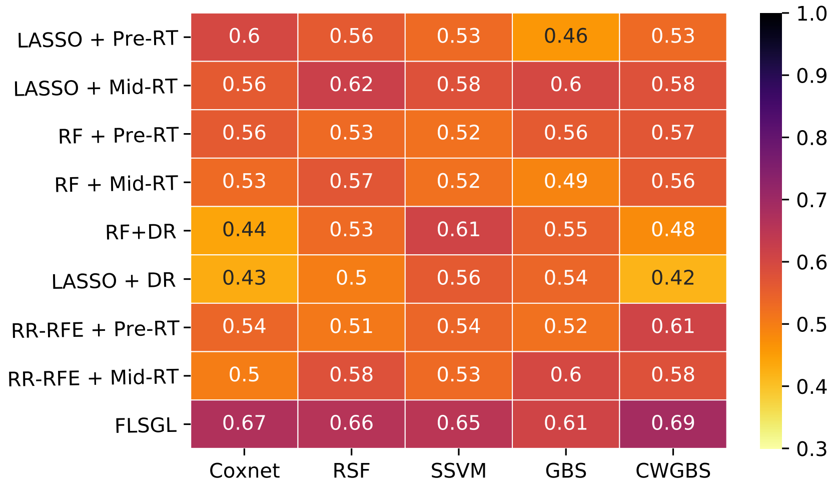

Figure S3. Heatmap of c-index values of overall survival prediction for different feature selection and survival analysis algorithms using FDG-PET radiomics. (DR: Delta Radiomics, Cox: Cox Net Survival Model, RR-RFE: Ridge Regression Recursive Feature Elimination, RF: Random Forest, FLSGL: Fused Laplacian Sparse Group Lasso, RSF: Random Survival Forest, GBS: Gradient Boosting Survival, SSVM: Survival Support Vector Machine, and CWGBS: Component-Wise Gradient Boosting Survival)

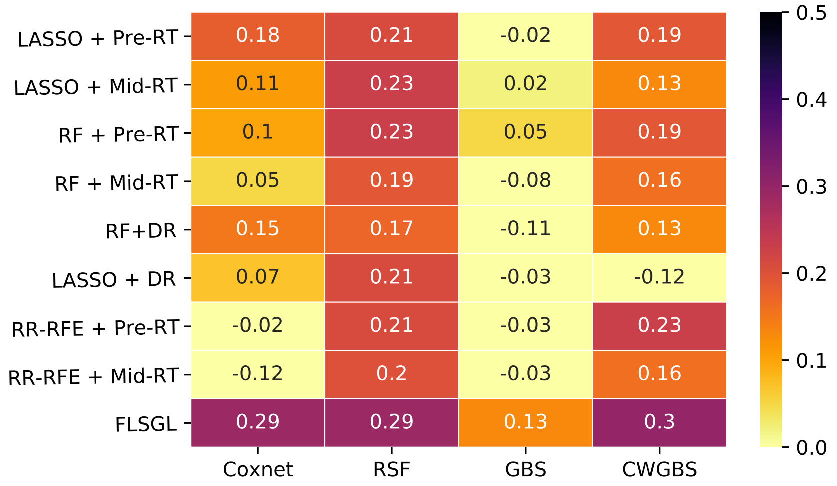

Figure S4. Heatmap of IPA values of overall survival prediction for different feature selection and survival analysis algorithms using FDG-PET radiomics. (DR: Delta Radiomics, Cox: Cox Net Survival Model, RR-RFE: Ridge Regression Recursive Feature Elimination, RF: Random Forest, FLSGL: Fused Laplacian Sparse Group Lasso, RSF: Random Survival Forest, GBS: Gradient Boosting Survival, and CWGBS: Component-Wise Gradient Boosting Survival)
